# Involvement of the Liver in COVID-19: A Systematic Review

**DOI:** 10.4269/ajtmh.21-1240

**Published:** 2022-02-24

**Authors:** Jayani C. Kariyawasam, Umesh Jayarajah, Visula Abeysuriya, Rishdha Riza, Suranjith L. Seneviratne

**Affiliations:** ^1^ Faculty of Medicine, Sir John Kotelawala Defence University, Ratmalana, Sri Lanka;; ^2^ Postgraduate Institute of Medicine, University of Colombo, Colombo, Sri Lanka;; ^3^ Nawaloka Hospital Research and Education Foundation, Nawaloka Hospitals, Colombo, Sri Lanka;; ^4^ Colombo South Teaching Hospital, Colombo, Sri Lanka

## Abstract

COVID-19, a respiratory viral infection, has affected 388 million individuals worldwide as of the February 4, 2022. In this review, we have outlined the important liver manifestations of COVID-19 and discussed the possible underlying pathophysiological mechanisms and their diagnosis and management. Factors that may contribute to hepatic involvement in COVID-19 include direct viral cytopathic effects, exaggerated immune responses/systemic inflammatory response syndrome, hypoxia-induced changes, vascular changes due to coagulopathy, endothelitis, cardiac congestion from right heart failure, and drug-induced liver injury. The majority of COVID-19-associated liver symptoms are mild and self-limiting. Thus management is generally supportive. Liver function tests and abdominal imaging are the primary investigations done in relation to liver involvement in COVID-19 patients. However, imaging findings are nonspecific. Severe acute respiratory syndrome coronavirus 2 RNA has been found in liver biopsies. However, there is limited place for liver biopsy in the clinical context, as it does not influence management. Although, the management is supportive in the majority of patients without previous liver disease, special emphasis is needed in those with nonalcoholic fatty liver disease, cirrhosis, hepatocellular carcinoma, hepatitis B and C infections, and alcoholic liver disease, and in liver transplant recipients.

## INTRODUCTION

COVID-19 is caused by severe acute respiratory syndrome coronavirus 2 (SARS-CoV-2) and as of November 28, 2021, there have been more than 260 million cases worldwide and over 5 million deaths. ^1^ Several vaccines have already been developed with a view to controlling this pandemic. There are two genera of human coronaviruses: alpha (human coronavirus [HCoV]-229E and HCoV-NL63) and beta (HCoV-HKU1, HCoV-OC43, severe acute respiratory syndrome coronavirus 1 [SARS-CoV-1] and Middle East respiratory syndrome coronavirus [MERS-CoV]). The coronaviruses HCoV-OC43, HCoV-HKU1, HCoV-229E, and HCoV-NL63 cause mild disease, whereas the SARS-CoV-1, MERS-CoV, and SARS-CoV-2 may potentially cause severe disease. ^2^^,^ ^3^ Outbreaks of SARS-CoV-1 and MERS-CoV infections occurred in 2002 and 2012, respectively. ^4^ Severe acute respiratory syndrome coronavirus 2 has 70% and 40% genetic sequence similarity with SARS-CoV-1 and MERS-CoV. ^5^ Although fever and respiratory symptoms predominate in coronavirus infections, a range of liver manifestations is seen in SARS-CoV-1, MERS, and SARS-CoV-2 patients. ^6^^,^ ^7^

Table 1 shows a summary of the liver findings in SAR-CoV-1, MERS, and SARS-CoV-2. ^6^^,^ ^8^^–^ ^17^ Hepatic impairment was seen in up to 60% of patients with SARS-CoV-1. The main laboratory findings of SARS-CoV-1 were moderate to a marked elevation of alanine transaminase (ALT), decreased serum albumin, and increased serum bilirubin levels. ^11^^,^ ^16^ Pathological findings included prominent mitosis, acidophilic bodies, and mild to moderate lobular inflammation. Severe acute respiratory syndrome coronavirus 1 induced liver injury was supported by the presence of viral RNA in liver tissue. ^16^ Autopsies of SARS-CoV-1 patients found large numbers of viral particles in hepatocytes and hepatic vascular endothelial cells. ^18^ Some patients with severe MERS-CoV had raised liver aminotransferase (ALT and aspartate transaminase [AST]) levels and hyperbilirubinemia. ^12^^,^ ^13^ A low albumin level on the day of diagnosis was a predictor of disease severity. ^12^ As with SARS-CoV-1, mild portal tract and lobular lymphocytic infiltration, moderate steatosis, and scattered calcification were observed in MERS-CoV infections. ^19^ The incidence of liver injury in severe COVID-19 cases (74.4%) was higher than that of patients with mild disease (43%). The incidence of liver injury in COVID-19-associated deaths was 58%. ^8^ In this review, we have outlined the important liver manifestations of COVID-19 and discussed the possible pathophysiological mechanisms and their diagnosis and management.

**Table 1:** Liver involvement of SARS-CoV-1, MERS, and SARS-CoV-2

	SARS-CoV-1	MERS-CoV	SARS-CoV-2
Incidence of liver injury	60% ^6^	60% ^6^	14.8–53% ^8^
Expression of entry receptor on cholangiocytes	NA	NA	ACE2 receptor expression is higher than on hepatocytes ^9^
Expression of entry receptor on hepatocytes	ACE2 receptor expression is abundant ^9^	DPP-4 receptor expression is high in liver ^10^	ACE2 receptor expression is low ^9^
Expression of entry receptor in Kupffer cells, liver endothelial cells, and other inflammatory cells	NA	NA	ACE2 receptor is expressed ^9^
Liver enzyme level	Mild to moderate elevation of ALT and AST—53% ^11^	Elevation of ALT and/or AST ^12^^,^ ^13^	Elevation of ALT 23.3% and AST 23.4% ^14^
Albumin level	Decreased serum albumin ^1^	Decreased levels of albumin ^12^^,^ ^13^^,^ ^15^	Decreased levels of albumin 61.3% ^14^
Bilirubin level	Increased serum bilirubin ^6^	Increased serum bilirubin ^12^^,^ ^13^	Increased serum bilirubin 27.9% ^4^
Serum GGT level	NA	NA	Increased in severe cases 27.9% ^14^
Pathological manifestations of liver injury	Antemortem Mild lobular activities with occasional acidophilic bodies and prominent Kupffer cell Mildly inflamed portal tracts with lymphocytic infiltration Nonspecific inflammation in the liver in biopsy Hydropic degeneration Steatosis Focal necrosis ^6^ Postmortem histopathological findings- Necrosis Nodular cirrhosis Minor inflammatory changes Hydropic and fatty degeneration Interstitial cell proliferation Mild fatty acid degeneration Mild congestion ^6^ Significant increase in mitotic cells with eosinophilic bodies and balloon-like hepatocytes ^15^	Postmortem histopathological findings Mild chronic lymphocytic portal and lobular inflammation Reactive parenchyma with mild cellular hydropic degeneration Rare multinucleated hepatocytes and mild disarray of the hepatic plates Mild sinusoidal lymphocytosis and small necroinflammatory foci in the hepatic lobules Congestion, hemorrhage, and focal perivenular loss of hepatocytes Macrovesicular perivenular steatotic change, sinusoidal congestion, hemorrhage, and focal perivenular loss of hepatocytes Scattered calcifications Nonspecific hepatitis ^6^	Postmortem histopathological findings Microvescicular steatosis Mild lobular and portal activity ^16^ Hepatomegaly Hepatocyte degeneration Lobular focal necrosis Neutrophil infiltration (lymphocytes and monocytes in portal area) Congestion of hepatic sinuses with microthrombosis Mild sinusoidal dilatation Mild lobular lymphocytic infiltration Patchy hepatic necrosis in the periportal and centrilobular areas Over activation of T cells ^17^

ACE2 = angiotensin-converting enzyme 2; ALT = alanine aminotransferase; AST = aspartate aminotransferase; DPP-4 = dipeptidyl peptidase 4; MERS-CoV = Middle East respiratory syndrome coronavirus; NA = not available/applicable; SARS-CoV-1 = severe acute respiratory syndrome coronavirus 1; SARS-CoV-2 = severe acute respiratory syndrome coronavirus 2.

## LITERATURE SEARCH

**Figure 1.**
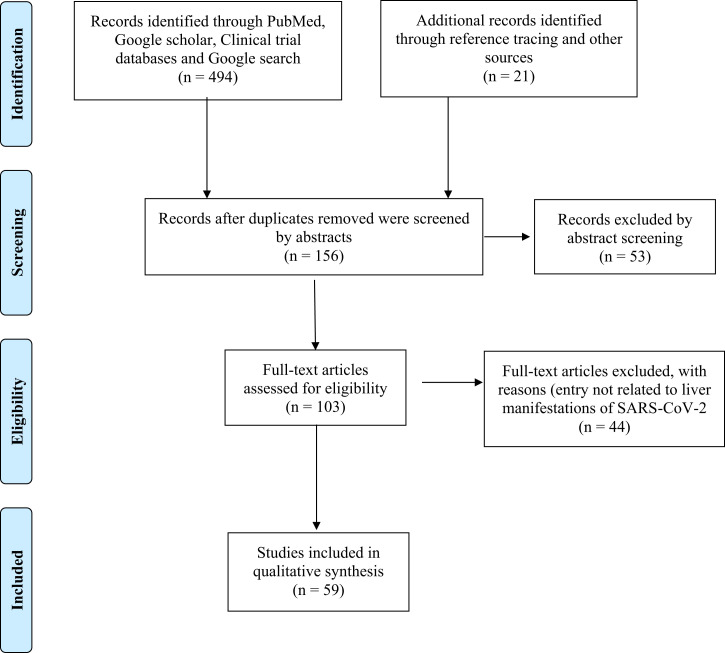
PRISMA flow chart. This figure appears in color at www.ajtmh.org.

We searched PubMed, Google Scholar, and Google from January 2020 to November 28, 2021, for articles written in English that describe the liver effects of COVID-19, using the search terms “coronaviruses and liver,” “COVID-19 and liver,” “COVID-19, and liver symptoms,” “COVID-19 and hepatic,” “COVID-19 and liver function tests,” “COVID-19 and liver inflammation,” “SARS-Cov-2 and liver,” and “transplantation during COVID-19.” Reference lists of the articles were scanned to identify any additional studies. The article title and abstract were read for the initial selection and then the full-text article was read. Reference lists of the full-text articles were scanned to identify any additional studies. All types of research articles, including original research articles, reviews, case series, short communications, and case reports were considered. Of the 103 articles identified, 59 were analyzed further (Figure 1).

### Liver-related outcomes associated with COVID-19.

The current literature has several studies on liver-related outcomes in COVID-19. However, the definition of liver injury tends to vary among the different studies. Furthermore, specifying liver-related outcomes in COVID-19 patients is made difficult because of the studies describing different etiologies, different disease severities, small numbers of study participants from a single geographical location, and the lack of correlation of liver test results with preexisting liver conditions. Preexisting chronic liver disease (CLD) may predispose a person to adverse outcomes following COVID-19 because of immune dysregulation. ^20^ Marjot’s international registry study found mortality to be high in cirrhosis patients (32%) compared with those without cirrhosis (8%). ^20^ Furthermore, a strong correlation between the stage of liver disease and the rate of intensive care unit (ICU) admissions, renal replacement therapy, and death was found. ^20^ The cause of death in patients with CLD/cirrhosis was respiratory related in the majority (71%) and 19% were liver related. ^20^ On admission, although respiratory symptoms were similar among the CLD and non-CLD individuals, gastrointestinal (GI) side effects were comparatively higher in CLD patients. ^20^ Furthermore, baseline liver disease stage and alcohol-related liver disease were risk factors for death from COVD-19. ^20^ In a multicenter cohort study by Lavarone et al., the mortality of those with cirrhosis was significantly higher than those without cirrhosis (34% versus 18%, respectively). ^21^ Further, the mortality associated with cirrhosis was higher than among those with cirrhosis and bacterial infection. ^21^ Another study by Marjot et al. ^22^ in autoimmune hepatitis patients found that autoimmune hepatitis (AIH) and immunosuppression were not significantly associated with death despite the use of medications that suppressed the immune system. This may be because of the low sample number (*N* = 77) of AIH patients.

## PATHOPHYSIOLOGY OF LIVER INVOLVEMENT IN COVID-19

**Figure 2.**
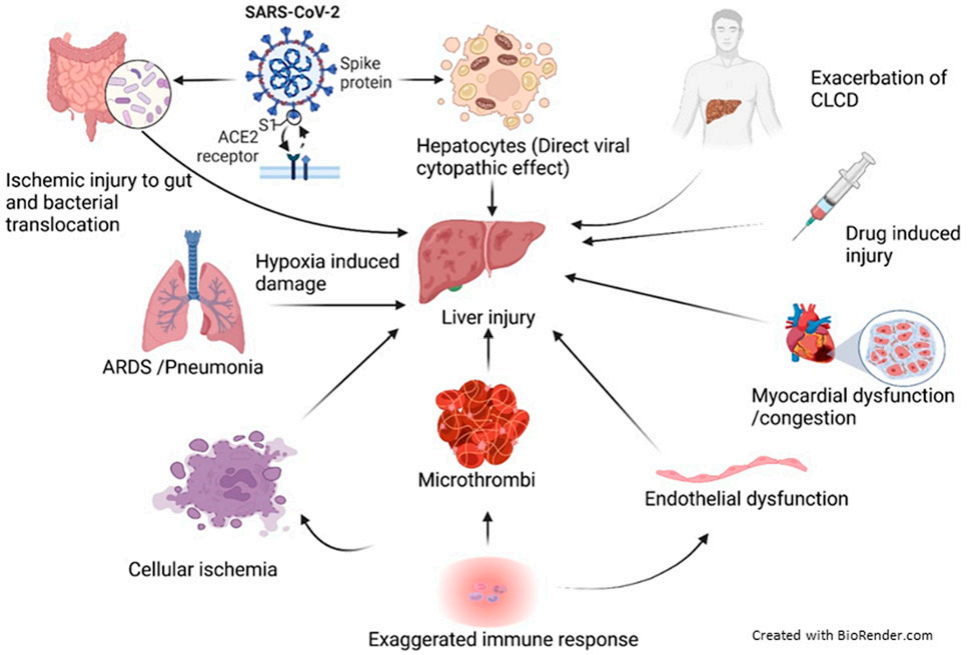
Pathophysiological processes that may lead to liver injury in COVID-19. Following SARS-CoV-2 infection, liver injury may result due to direct cytopathic effects (due to viral entry through ACE2 receptors on hepatocytes and cholangiocytes) or hypoxia-induced damage (resulting from ARDS or pneumonia-associated hypoxia) or bacterial translocation and inflammation (direct viral injury and ischemic injury of the gut or disruption of gut–mucosal barrier) or systemic hypotension and cellular ischemia, abnormal coagulation/microthrombi, endothelial dysfunction (resulting from exaggerated immune responses/systemic inflammation) or cardiac congestion from right heart failure (due to myocardial dysfunction), or drug-induced liver injury or exacerbation of chronic liver disease. SARS-CoV-2 = severe acute respiratory syndrome corona virus 2; ACE2 = angiotensin-converting enzyme 2; ARDS = acute respiratory distress syndrome; CLCD = chronic liver cell disease. This figure appears in color at www.ajtmh.org.

Factors that may contribute to liver involvement in COVID-19 include direct viral cytopathic effects, exaggerated immune responses/systemic inflammatory response syndrome (SIRS), hypoxia-induced changes, vascular changes due to coagulopathy, ^23^ endothelitis, cardiac congestion from right heart failure, and drug-induced liver injury. ^8^^,^ ^24^ These factors may also exacerbate any underlying liver disease. The pathophysiological processes involved in liver impairment in COVID-19 are summarized in Figure 2.

### Angiotensin-converting enzyme 2 receptors.

Angiotensin-converting enzyme 2 (ACE2) receptors provide a gateway for viral entry, and its tissue distribution determines the pattern of viral tropism. There is high expression of ACE2 on cholangiocytes (epithelial cells of the bile duct) and low expression on hepatocytes, Kupffer cells (liver macrophages), and endothelial cells. ^9^ Levels of expression on bile ducts are similar to type II alveolar cells. ^25^ Cholangiocytes undergo syncytia formation following SARS-CoV-2 infection and similar observations have been noted when the virus infects adult human cholangiocyte organoids. The virus is able to replicate within the bile duct epithelium. Levels of ACE2 expression may be affected by many factors. Preexisting liver disease, hypoxia, drug-induced liver injury, and inflammation increase the levels of expression ^24^ and may, in turn, enhance viral-induced cytotoxicity. In vitro studies found pretreatment of ACE2 receptors with trypsin increases the binding affinity of SARS-CoV-2 spike protein. Liver epithelial cells express trypsin, and this may facilitate viral entry despite low ACE2 expression levels. Furthermore, the spike protein of SARS-CoV-2 has a furin-like proteolytic site. As furin is predominantly expressed in the liver, it may support viral entry. Cell line studies have found viral entry to depend on the PIKfyve-TCP2 endocytotic pathway that is expressed in the liver and gall bladder, at comparable levels to the lung.

### Direct viral cytotoxicity.

The renin-angiotensin system (RAS) plays a major role in liver inflammation, tissue remodeling, and fibrosis. Angiotensin-converting enzyme 2 is a key negative regulator of the RAS and limits fibrosis through the degradation of Angiotensin II and the formation of Angiotensin (1–7). Upon binding of the SARS-CoV-2 virus, ACE2 is endocytosed and levels are reduced on the cell surface. Murine studies found reduced ACE2 levels to worsen liver fibrosis in chronic liver injury models. ^26^ Direct viral cytotoxicity gives rise to steatohepatitis by interfering with lipogenesis and in turn, may worsen chronic liver diseases such as nonalcoholic fatty liver disease (NAFLD) and alcoholic hepatitis. ^24^

### Immune-mediated effects.

An exaggerated inflammatory response in COVID-19 leads to lymphocyte activation, neutrophilia, and an increase in C-reactive protein (CRP) and inflammatory cytokines. Levels of serum interleukin (IL)-2, IL-6, IL-7, IL-10, tumor necrosis factor (TNF)-α, granulocyte-macrophage colony-stimulating factor (GM-CSF), interferon-inducible-protein-10, monocyte chemotactic protein-1, and macrophage-inflammatory-protein-1 alpha are significantly higher in severe COVID-19. ^9^^,^ ^27^ A CRP ≥ 20 mg/L and a lymphocyte count < 1.1 × 10^9^/L are independent risk factors for liver injury. Lymphopenia is noted in 63–70.3% of COVID-19 patients. Postmortem liver histology shows microvesicular steatosis and T cell accumulation, pointing to the presence of immune-mediated damage. ^28^ The systemic inflammatory response secondary to the infection causes systemic hypotension, cellular ischemia, abnormal coagulation, microthrombi, and endothelial dysfunction and may further exacerbate the liver damage caused by direct viral cytopathic effects. Thus liver damage should be suspected and treated promptly in a clinically deteriorating patient with systemic manifestations of COVID-19.

### Hypoxia-related effects.

Liver hypoxia (because of microvascular thrombosis and gas exchange defects secondary to lung injury) may cause hepatic damage. Ischemic injury to the gut with resulting intestinal endotoxemia, and activation of the sympathetic nervous and adrenocortical systems may further contribute to liver damage. ^9^^,^ ^29^ Furthermore, COVID-19-induced myocardial dysfunction can potentially give rise to right heart failure, adding to the existing damage, and worsening ischemic liver injury. Elevated transaminases in the context of respiratory failure, shock, and heart failure in severe COVID-19 may be indicators of this pathophysiological mechanism. ^30^

### Drug-related cytotoxicity.

As most COVID-19 patients have fever, antipyretics containing acetaminophen are frequently used. Higher doses of this medication are known to cause liver damage. Many antiviral drugs are administered (alone or in combination) and some of them may have adverse effects on the liver (Table 2). ^31^^–^ ^48^ It should be noted that some of the medications are no longer in use for COVID-19 in current clinical practice. Lopinavir/ritonavir increases the odds of liver injury by fourfold. Thus close monitoring is needed in such patients especially when abnormal liver function tests (LFTs) have been observed at admission. ^49^

**Table 2:** Potential liver side effects of currently and previously used medications in COVID-19

Class	Drug	Dosage	Administration	Liver side effects	References
Antivirals	Remdesivir (In phase 3 clinical trials)	Loading dose 200 mg over 30–120 minutes on day 1 followed by 100 mg once daily for remaining 4/9 days Not needing invasive mechanical ventilation/ECMO: for 5 days Needs mechanical ventilation or ECMO for 10 days	Intravenous	1–10%—liver enzyme derangement, hyperbilirubinemia	^31^^,^ ^32^^,^ ^33^^,^ ^34^
	Paxlovid ((PF-07321332 150 mg and ritonavir 100 mg)	300 mg PF-07321332 (two 150 mg tablets) with 100 mg ritonavir (one 100 mg tablet) all taken together orally every 12 hours for 5 days	Oral	May cause liver damage because of ritonavir. No dosage adjustment is needed for patients with either mild (Child-Pugh Class A) or moderate (Child-Pugh Class B) hepatic impairment.	^47^
	Molnupiravir	800 mg (administered as four 200 mg capsules) taken orally every 12 hours with or without food for 5 days	Oral	N/A	^48^
	Lopinavir/ ritonavir (LPV/r) (Kaletra)	400/100 mg twice daily or 800/200 mg once daily for 14 days.	Oral (administer with or without food)	1–10%—hepatic disorders, cholangitis, hyperbilirubinemia	^35^
	Ribavirin (In phase 2 clinical trials)	400 mg twice daily for 14 days (in clinical trials)—dosing not defined	Oral (administer with food)	0.1–1%—Hepatic disorders Less than 0.1%—Cholangitis, hepatic failure	^36^
	Darunavir	1 pill of DRV/c (a single-tablet regimen containing 800 mg of darunavir and 150 mg of cobicistat) per day for 5 days	Oral	Moderate to severe elevations in serum aminotransferase levels (> 5 × ULN) in 3–10% of patients overall	^37^
	Favipiravir	1,800 mg twice daily on day 1 followed by 800 mg twice daily on days 2 to a maximum of 14 days	Oral	Liver enzyme derangement (2%)	^38^
Immunomodulatory drugs	Tocilizumab	4–8 mg/kg (maximum 800 mg) over 1 hour; or 400 mg once Consider an additional dose 8–12 hours later if continued clinical deterioration (maximum of 2 doses)	Intravenous	Frequency not known—Hepatic disorders	^31^
	Interferon α/β	INF-β-1b 0.25 mg alternated for 3 days (in clinical trial)—dosing not established	Subcutaneous injection	0.1–1%—Hepatic disorders, autoimmune hepatitis	^31^
	Baricitinib (completed clinical trial)	4 mg once daily	Oral	Frequency not known—Abnormal liver enzymes	^39^^,^ ^40^
		Baricitinib + antiviral therapy administration for 2 weeks			
	Imatinib	400 mg daily for 14 days	Oral	Common elevations in serum aminotransferase levels mild elevations in serum bilirubin can occur. These abnormalities are usually mild, asymptomatic, and resolve despite continuing therapy. Linked to rare instances of clinically apparent acute liver injury with jaundice.	^41^^,^ ^42^
Antiparasitic	Chloroquine	500 mg twice/day for 10 days.	Oral (administer with food)	Less than 0.1%—Hepatitis	^( 33^
	Hydroxychloroquine	Loading dose of 400 mg twice daily for 1 day, followed by 200 mg twice daily for 4 days.	Oral (administer with food)	Frequency not known—Acute hepatic failure	^31^^,^ ^43^
Steroids	Dexamethasone	6mg daily for 7–10 days	Oral	Frequency not known—Acute hepatic failure	^44^^,^ ^45^
Antibiotic	Azithromycin	NA	NA	Low rate of acute, transient, and asymptomatic elevation in serum aminotransferases which occurs in 1–2% of patients treated for short periods, and a somewhat higher proportion of patients given azithromycin long term. Rarely cause clinically apparent liver injury.	^46^

ALT = alanine transaminase; ECMO = extracorporeal membrane oxygenation; INF-β = interferon-beta; LPV/r = lopinavir/ritonavir; NA = not applicable.

### Gut microbiota.

Recent studies on gut microbiota have suggested an alteration in intestinal microbiota composition (i.e., dysbiosis) contributes to different immune-mediated inflammatory diseases. ^50^ Similarly, in COVID-19, gut microbiota dysbiosis might play an important role in determining the clinical outcome of patients with underlying comorbid conditions such as diabetes, hypertension, and obesity. ^51^ For instance, gut microbiota diversity is generally decreased in older individuals and COVID-19 is also more severe and fatal in this group of individuals raising a potential role of the gut microbiota in overall pathogenesis and outcomes. ^52^ Furthermore, it has been suggested that COVID-19 patients are depleted of gut bacteria with known immunomodulatory potential. ^53^ Additionally, inflammation induced by gut dysbiosis represents an important factor in cardiometabolic and diabetic pathogenesis and may contribute to increasing the severity of COVID-19 in the most vulnerable patients. ^54^ As diet plays a critical role in modulating the gut microbiota, there has been increased interest in evaluating the health benefits and disease-preventing properties of diet and dietary habits and their association with favorable patient outcomes. ^55^^,^ ^56^ The GI blood supply drains to the liver by the portal venous system. Thus disruption of the gut microbiota, with breach of the gut–mucosal barrier may lead to sepsis-induced hepatic dysfunction.

### Mitochondrial damage.

Preliminary observations suggest that SARS‐CoV‐2 affects mitochondrial activity. ^57^ Furthermore, Wang et al. identified mitochondrial crista abnormalities in liver specimens from COVID‐19 patients. Interestingly, impaired mitochondrial activity has also been implicated in the pathogenesis of NAFLD/non-alcoholic steatohepatitis. ^58^ Thus, SARS‐CoV‐2 infection might worsen the metabolic state and aggravate preexisting NAFLD by these mechanisms.

## HEPATIC MANIFESTATIONS IN COVID-19

The COVID-19-associated liver injury is defined as liver damage occurring due to the virus or its treatment in those with or without preexisting liver damage. ^59^ Several biochemical definitions for liver injury have been proposed. These include, ALT or AST exceeding three times the upper limit of normal, and alkaline phosphatase (ALP), gamma-glutamyl transferase (GGT), or total bilirubin exceeding two times the upper limit of normal. The overall incidence of liver damage due to COVID-19 varies from 14.8% to 53%, ^24^ and is more frequent in severe than in mild disease. The degree of liver injury is generally mild ^60^ and predominantly hepatocellular rather than cholestatic. ^61^ Those with GI symptoms were more prone to developing liver involvement. ^62^ Li and Xiao ^63^ classified liver involvement in COVID-19 into two types—specific and nonspecific. The specific type caused three or higher and two or higher fold elevations in ALT/AST and total bilirubin levels, respectively. The nonspecific type caused mild and transient LFT abnormalities, was due to general inflammation, and usually does not need any special treatment. ^60^ Hepatic injury is commonly associated with decreased lymphocyte counts, raised neutrophil counts, and male gender. ^64^ This reflects the role of innate immunity/inflammation in COVID-19-associated hepatic injury. More studies are needed to support the relationship between male gender and hepatic injury. The highest ALT, AST, APT, and GGT levels are significantly associated with high body temperatures during the illness. ^65^ This suggests that changes in the body temperature may contribute to the pathophysiology of COVID-19-associated liver disease. The presence of hepatic injury has been associated with the development of acute respiratory distress syndrome (ARDS). Larger cohort studies should help to better understand this process. Acute liver injury is associated with high mortality. This fulminate hepatic failure may result from direct viral replication or increased inflammation. A summary of the liver-related investigations findings and the treatments used are shown in Table 3. ^28^^,^ ^49^^,^ ^66^^–^ ^80^

**Table 3:** Clinical characteristics, liver manifestations, and treatments of patients with COVID-19

First author, year, and country	Article type	Total no of patients (had GI symptoms)	No of males *n* (%)	Average age (Years)	No of patients had livers abnormalities before or after COVID-19 (%)		Increase of aminotransferase levels U/L, mean/median (*n*)		Increase of bilirubin mg/dL, *n* (%)	Decrease of albumin g/L	Alkaline phosphatase U/L, mean ± SD (*n*)	Gamma-glutamyl transferase U/L (%)
					Before	After	ALT	AST				
Beigmohammadi, 2020 ^66^ (Iran)	RA	7	5 (71%)	67.85	1 (peptic ulcer disease)	NA	NA	NA	NA	NA	NA	NA
Cardoso, 2020 ^67^ (Portugal)	RL	20	18 (90%)	67	18	0	(≤ 55 IU/L) On admission— 31 U/L Peaked on ICU day 8–82	(≤ 34 IU/L) On admission— 51 U/L Peaked on ICU day 5—69 U/L	(≤ 1.2 mg/dL) On admission— 0.65 mg/dL Peaked on ICU day 3–1.16 mg/dL	(Normal 40.0– 55.0 g/L) 31.6 g/L 97 (98%)	NA	NA
Cai, 2020 ^49^ (China)	RA	417	198 (47.5%)	47- M	21 (5.04%) (NAFLD, alcoholic liver disease, and chronic hepatitis B)	396 (95%)	On admission 27 U/L—AbLT 47 U/L—LI During hospitalization 69 U/L—nonsevere 79 U/L—severe	On admission 34 U/L—AbLT 47.2 U/L—LI During hospitalization 34 U/L—nonsevere 58 U/L—severe	On admission 16.8 μmol/L—AbLT 17.2 μmol/L—LI During hospitalization 19 μmol/L—nonsevere 22 μmol/L—severe	NA	NA	On admission 36.45 U/L—AbLT 134.91 U/L—LI During hospitalization 40 U/L—nonsevere 92 U/L—severe
Chen N, 2020 ^68^ (China)	RA	99	67 (68%)	55.5	0	NA	(U/L; normal range 9.0–50.0) 39 U/L 28 (28%)	(U/L; normal range 15.0–40.0 34 U/L 35 (35%)	(μmol/L; normal range 0.0–21.0) 15.1 μmol/L (7.3%) 18 (18%)	(g/L; normal range 40.0–55.0) 31.6 g/L (4.0%) 97 (98%)	NA	NA
Guan W-J, 2020 ^69^ (China)	RA	1,099	601 (55%)	NA	23 (2.3%) Hep B	NA	> 40 U/L 168/757 (22.2%)	> 40 U/L 158/741 (21.3%)	> 17.1 μmol/L 76/722 (10.5%)	NA	NA	NA
Effenberger, 2020 ^70^ (Austria)	RA	32	NA	73.5—with liver damage 69.9—no liver damage	3 (9.4%) hepatitis C and successful antiviral therapy or signs of MAFLD	NA	No liver damage— 31.3 U/L Liver damage— 76.3 U/L	No liver damage— 19.5 U/L Liver damage— 67.1 U/L	NA	NA	NA	NA
Ji D, 2020 ^28^ (China)	RA	140	82 (58.6)	41.9	54 (38.6%) had NAFLD 7 (5.0%) had positive HBsAg	22 (15.7%) had CLD (3— cirrhosis 6—CHB 13—NAFLD)	Non—CLD *n* (%) 59 (50.0) CLD—15 (68.2)	Non—CLD *n* (%) 19 (16.1) CLD—6 (27.3)	Non—CLD *n* (%)—7 (5.9) CLD—2 (9.1)	NA	Non—CLD *n* (%)— 4 (3.4) CLD—0 (0)	NA
Jin X, 2020 ^71^ (China)	RA	651 (74%)	37 (50%)	46.14	8 (10.8%) Chronic liver disease	NA	(U/L; normal range 9–50) With GI symptoms— 25.0 Without GI symptoms—21.5	(U/L; normal range 15–40 With GI symptoms—29.35 Without GI symptoms—24.4	(umol/L; normal range 0–26) With GI symptoms— 10.0 Without GI symptoms—9.6	(g/L; normal range 40–55) with GI symptoms— 40.13 g/L Without GI symptoms— 41.50 g/L	NA	NA
Lin, 2020 ^72^ (China)	RA	95 (58%)	45 (47%)	49.5	0	NA	(U/L; normal range 7–40 in female, 9–50 in male) Initial—0 During hospitalization— 22 (23.2%)	(U/L; normal rage 13–35 in female, 15–40 in male) Initial—1 (1.1) During hospitalization— 4 (4.2)	(μmol/L; normal range 3.0–24.0) During hospitalization— 22 (23.2)	NA	NA	NA
Luo, 2020 ^73^ (China)	RA	1,141 (183%)	102 (55.7%)	53.8	NA	NA	(Normal range 9–50 U/L) 66.4%	(Normal range 15–40 U/L) 65.8%	NA	NA	NA	NA
Mo, 2020 ^74^ (China)	RA	155	86 (55.5%)	54	NA	NA	23 U/L	32 U/L	NA	38 g/L (34–41)	NA	NA
Pan, 2020 ^75^ (China)	RA	204 (103)	107 (52.5%)	52.9	7 (3.4%) digestive disease	NA	Without digestive symptoms— 29.53 mmol/L With digestive symptoms— 42.24 mmol/L	Without digestive symptoms—27.48 With digestive symptoms— 35.12	NA	Without digestive symptoms— 35.84 g/L With digestive symptoms— 36.16 g/L	NA	NA
Singh and Khan, 2020 ^76^ (USA)	RA	2780	1,070 (38.5%)	5 LD group— 55.2 Non-LD—51.6	NA	NA	With liver disease— 100 U/L (130) Without liver disease— 80 UL (70)	With liver disease— 221 U/L (130) Without liver disease— 133 U/L (770)	With liver disease— 1.2 mg/dL (120) Without liver disease— 0.8 mg/dL (770)	With liver disease— 2.6 (120) Without liver disease— 2.5 (770)	With liver disease— 153 U/L (120) Without liver disease— 93 U/L (770)	With liver disease— 278 U/L (10) Without liver disease— 99 U/L (30)
Xu X-W, 2020 ^77^ (China)	RA	62	36 (58%)	41	7 (11%)	NA	22 U/L	10 (16%) 26 U/L	NA	NA	NA	NA
Xu Y, 2020 ^78^ (China)	BC	10	6 (60%)	12	NA	NA	One patient had above normal (9–50 U/L)	Two patients had above normal (5–60 U/L)	NA	All within normal range (40–55 g/L)	NA	NA
Zhang H, 2020 ^79^ (China)	RA	505 (164)	228 (45.1%)	51.2	NA	NA	(Normal range 7–40 U/L) 42.1 U/L	(Normal range 13–35 UL) 48.1 U/L	NA	NA	NA	(Normal range 7–45 U/L) 45.8 U/L
Zhou, 2020 ^80^ (China)	RA	191	119 (62%)	56	NA	NA	> 40 U/L 59/189 (31%)	NA	NA	32·3 g/L	NA	NA

AbLT = abnormal liver tests; ALT = alanine aminotransferase; AST = aspartate aminotransferase; CLD = chronic liver disease; CRRT = continuous renal replacement therapy; ECMO = extracorporeal membrane oxygenation; HBsAg = hepatitis B surface antigen; ICU = intensive care unit; LI = liver injury; MAFLD = metabolic associated fatty liver disease; NA = not available; NAFLD = nonalcoholic fatty liver disease; NSAIDs = nonsteroidal anti-inflammatory drugs; PaO_2_/FiO_2_ = ratio of arterial oxygen partial pressure to fractional inspired oxygen.

### Elevation in aminotransferase levels.

The commonest liver test abnormalities reported are mild to moderate elevations in ALT and AST levels (seen in 14–53% of cases). ^81^^,^ ^82^ Significant elevations of ALT and AST are commoner in those with other digestive symptoms. ^83^ Raised liver aminotransferases are associated with significantly longer hospital stays. ^84^^,^ ^85^ A multicenter retrospective cohort study of 5,771 adult COVID-19 patients found AST to increase initially followed by ALT. Aspartate transaminase abnormalities were associated with the highest risk of mortality. ^64^ Although ALT is more specific to the liver, higher AST levels may be associated with injury to other organs or because of mitochondrial injury and should thus be interpreted with caution.

### Elevations in bilirubin levels.

Elevated bilirubin levels are observed in 20–40% of patients and 10% had very high levels. ^63^^,^ ^69^ Bilirubin levels are significantly higher in those with severe disease and are associated with a poorer prognosis. ^86^ Most of the studies do not indicate whether the hypebilirubinemia is of the direct or indirect type. A study from Spain found a biphasic pattern of hyperbilirubinemia, initially hepatocellular and later cholestatic in type. ^87^ This suggests that the elevation of bilirubin may be because of both direct hepatic injury and cholestasis. In addition to the increase in serum total bilirubin levels, raised conjugated bilirubin levels and conjugated to unconjugated bilirubin ratios were observed in COVID-19 patients. The high bilirubin levels may also be related to hemolysis. ^88^ Further studies would delineate the predominant pathogenesis of elevated bilirubin levels in COVID-19.

### Reduced synthetic function.

Up to 4% of patients with severe COVID-19 had reduced albumin levels. ^69^ Studies have found lower albumin levels to be associated with a poorer prognosis (severe pneumonia, longer hospital stays, and higher mortality). ^8^^,^ ^87^^,^ ^89^ This may be due to a direct effect of the virus on the liver or due to systemic inflammation in severe COVID-19. The low albumin levels may be due to switching off of albumin production by the liver, increased catabolism or loss of protein through the GI tract during COVID-19. Thus, the low albumin level mentioned in the studies should not be considered a direct marker of reduced liver function. As histological data do not suggest severe hepatic injury, it would be unlikely that low albumin is mainly contributed by hepatic dysfunction. Prothrombin time (PT) has been suggested as a predictive factor for clinical outcomes in COVID-19 patients. The survival rate is significantly lower in patients with prolonged PT. ^90^ Baranovskii et al. found significantly prolonged admission PT in ICU-transferred patients compared with stable COVID-19 patients. ^91^ Such findings may be due to systemic inflammation–related coagulopathy rather than reduced hepatic function.

### Raised gamma-GT levels.

Elevations of serum GGT levels point to the presence of cholangiocyte injury ^81^^,^ ^92^ and are observed in a sizeable proportion of those with severe COVID-19. Elevation of GGT in association with a rise in ALP would suggest cholestasis. The need for ICU care and reduced survival was observed in COVID-19 patients with a cholestatic pattern of hepatic injury. ^24^^,^ ^93^

### Pathological changes on liver histology.

The described pathological changes in liver histology are mainly ascertained from postmortem studies. Most of the studies do not indicate whether the patients had preexisting liver disease or the severity of the liver derangement, precluding useful interpretation of the pathological findings. The liver histology changes noted in COVID-19 include moderate microvascular and macrovascular steatosis and mild lobular portal inflammation. ^17^^,^ ^83^ In autopsy studies, centrilobular steatosis was seen, ^94^ with significant increases in mitotic cells, eosinophils, and balloon-like liver cells. ^16^ Lagana et al. found lobular necroinflammation (50%), portal inflammatory infiltrates (50%), cholestasis (38%), lobular apoptosis (25%), and macrovesicular steatosis (75%). ^95^ However, again the presence of preexisting liver disease or severity of the liver disease was not considered. A study by Wang et al, where the preexisting liver disease was excluded, found viral structures within hepatocytes by electron microscopy and raised the possibility of a direct cytopathic effect of the virus. ^79^

## MANAGEMENT OF LIVER INVOLVEMENT IN COVID-19

The majority of patients with COVID-19 have no or mild liver function abnormalities during the illness. ^96^^,^ ^97^ In mild COVID-19, hepatic damage may be transient and generally returns to normal without any special measures. ^63^^,^ ^96^ Thus, management is generally supportive with monitoring of LFTs. A summary of the liver management and recommendations are given in Table 4. ^98^^–^ ^110^

**Table 4:** Management of liver disease in COVID-19

	Investigations		Pharmacological management	Nonpharmacological management	Recommendations
	Laboratory and other biochemistry	Imaging and biopsy			
No preexisting liver disease	LFTs on admission (baseline) and at least twice weekly during hospital stay ^100^ Screen for HBV if systemic immunosuppression and tocilizumab has been given for > 7 days ^73^	Indicated only in suspicion of vascular or biliary disease ^96^^,^ ^97^ Limited/no place for liver biopsy ^102^	In moderate to severe liver injury lopinavir-ritonavir, tocilizumab are contraindicated ^102^	N/A	Baseline LFTs should be performed on admission to identify preexisting liver disease Transient elevation in LFTs may be seen and needs monitoring at least twice weekly in patients receiving hepatotoxic medication
Preexisting liver disease (NAFLD, cirrhosis, HCC, chronic Hepatitis B, alcoholic liver disease)	LFTs on admission (baseline) and at least every other day during hospital stay ^103^	Indicated only in suspicion of vascular or biliary disease ^101^ Limited/no place for liver biopsy ^102^	Concomitant administration of **tenofovir derivatives with lopinavir-ritonavir**—> increases tenofovir concentrations ^104^ Caution in use of **Paxlovid** (combination of Ritonavir + Nirmatrelvir) in preexisting liver disease, liver enzyme abnormality or liver inflammation ^110^ **Paxlovid** may induce hepatic enzymes and breakdown of nirmatrelvir or ritonavir ^110^ **Paracetamol** > 2 g per day to be avoided ^104^ **NSAIDs** used with caution ^104^ **Corticosteroids** used with caution in hepatitis B as they may increase the risk of hepatitis in chronic HBV ^104^ Continue treatment of HBV even during treatment of COVID-19, discontinuation of antiviral treatment of hepatitis B discouraged ^105^ **Anti-HBV drugs** may be considered when patients are on immunosuppressive treatment with careful monitoring ^105^	Strict measures to minimize exposure to COVID-19 especially in HCC due to very high risk of hospital-acquired COVID-19. ^98^ COVID-19 vaccines as early as possible ^109^ Treat HCC without delay. ^98^ Pneumococcal and influenza vaccines irrespective of the age. ^99^	Apart from HCV without decompensated cirrhosis, all other preexiting liver diseases should be managed as before. Use of immunosuppression in chronic liver disease requires caution and close monitoring. HCC should be treated without delay taking all precautions.
Liver transplant	Test donor and recipient for COVID-19 preoperatively	No specific recommendations	**Remdesivir**—risk of hepatotoxicity *Increased* levels with liver enzyme inducers ^108^ **Tocilizumab**—minor interaction with cyclosporine, tacrolimus, and sirolimus May reduce concentrations of calcineurin inhibitors. Use with chloroquine and hydroxychloroquine may produce additive toxicity. Myelosuppressive effect may potentiate hematological toxicity of ribavirin and interferon-beta. ^102^	Liver transplantation should not be postponed during pandemic ^106^ COVID-19 vaccines as early as possible ^109^	Although challenging, LT should not be postponed due to COVID-19 Early COVID-19 vaccination with third booster dose 1–2 months after second dose Perform baseline LFT before starting remdesivir therapy and monitor during therapy. Discontinue infusions if ALT and AST > 10 times ULN.

ALT = alanine aminotransferase; AST = aspartate aminotransferase; HBV = hepatitis B virus; HCC = hepatocellular carcinoma; HCV = hepatitis C virus; LFT = liver function test; LT = liver transplant; NAFLD = nonalcoholic fatty liver disease; NSAIDs = nonsteroidal anti-inflammatory drugs; ULN = upper limit of normal.

### Diagnostic aspects.

Liver function tests and abdominal imaging are the primary investigations done in relation to liver involvement in COVID-19 patients. Liver biochemistry including the liver enzymes (ALT and AST), serum bilirubin, albumin, and PT should be monitored for diagnosing liver damage. ^61^ However, the reasons for derangement of these blood tests are multifactorial and systemic inflammatory response because of COVID-19 may play a greater role than liver injury. Nevertheless, the liver tests should be performed during admission to establish a baseline and also to identify patients with suspected or known underlying liver disease. Further biochemical investigations may be needed in patients with known liver disease for example, known hepatitis B, C, and so on. The optimal interval for undertaking LFTs is uncertain. It has been suggested that LFTs be monitored at least twice weekly in COVID-19 patients receiving potential liver-toxic medications, whereas those with abnormal LFT results or with preexisting liver disease should be monitored more frequently. ^100^ Increased serum AST and lactate dehydrogenase (LDH) with normal ALT levels should raise the suspicion of alternative diagnoses such as skeletal muscle or myocardial injury. Abnormal LFTs are frequently noted at admission before antiviral treatment of COVID-19 is commenced. Abnormal LFTs at the onset of a COVID-19 infection may indicate underlying chronic liver disease (CLD) ^61^ and the treating physicians should take this into account. ^30^ The imaging modalities include abdominal ultrasonography and computed tomography scans. The imaging findings are nonspecific and are usually indicated when there is suspicion of portal venous thrombosis or biliary obstruction.

Lei et al. found liver hypo-echogenicity (homogeneous or heterogeneous) and peri-cholecystic fat stranding to be common positive findings on abdominal computed tomography. However, such findings are only detected in a subset of patients with liver derangement and hence such investigations should be used very selectively. ^101^ Postmortem liver biopsy often shows moderate microvascular steatosis and mild lobular and portal activity, but these are not specific for COVID-19. Therefore, there is limited or no place in liver biopsy in a clinical context. Although clues about the underlying pathological processes may be obtained, the influence of such findings on clinical management would be limited. ^102^

The American Association for the Study of Liver Diseases (AASLD) recommends the consideration of causes unrelated (e.g., hepatitis B virus [HBV] or hepatitis C virus [HCV]) to COVID-19 and other causes (e.g., myositis, ischemia, and cytokine release syndrome) for any liver test abnormalities. ^102^ In areas where viral hepatitis is prevalent, serological investigations for viral hepatitis may be considered depending on the clinical circumstances. In regions where it is less prevalent, monitoring of hepatic functions would suffice and further investigation for causes, may be restricted to cases where the hepatic functions do not normalize within a reasonable time frame (such as 2–3 months of its first detection). ^111^ The choice of evaluation for patients with persistent liver function abnormalities should include investigation for chronic parenchymal liver diseases and other infective causes and is based on the clinical presentation.

### Specific management in patients without previous liver diseases.

Liver functions should be monitored in COVID-19 patients at admission and during hospitalization. ^103^ The presence of abnormal liver tests is not a contraindication for investigational or off-label treatment of COVID-19. ^102^ However, such patients should be closely monitored while receiving any antivirals and off-label agents with potential liver toxicity. Known liver-toxic medications such as lopinavir-ritonavir and tocilizumab should be withheld if there is moderate to severe liver injury. If systemic immunosuppression such as corticosteroids and tocilizumab is administered for more than 7 days, screening for the HBV is recommended especially in regions where this infection is prevalent. ^111^ Drug interactions need to be considered when prescribing for COVID-19 patients with chronic liver disease. Paracetamol (doses > 2 g per day to be avoided in patients with chronic liver disease) and nonsteroidal anti-inflammatory drugs NSAIDs should be used with caution. The use of corticosteroids in COVID-19 may increase the risk of hepatitis in chronic HBV patients. Thus, one should be cautious with its use and if used done with close monitoring. Certain combinations of drugs are best avoided in patients with preexisting liver disease. For example, concomitant administration of tenofovir derivatives with lopinavir–ritonavir is relatively contraindicated as the concentration of tenofovir may be increased due to a drug interaction. In such cases, suitable alternatives should be used. ^104^

## COVID-19 IN PATIENTS WITH DIAGNOSED LIVER DISEASES

Those with preexisting liver disease, the elderly, or individuals who consume high amounts of alcohol or are obese should be monitored closely. The AASLD recommends the consideration of causes unrelated to COVID-19 (e.g., HBV or HCV) and other causes (e.g., myositis, ischemia, and cytokine release syndrome) for any observed liver test abnormalities. Patients receiving immunosuppressive medications (liver transplant recipients or those with autoimmune hepatitis) should be managed as they were prior to the pandemic. Those with chronic HBV should continue their treatment and in HCV patients without decompensated cirrhosis treatment may be delayed. Hepatocellular carcinoma should be treated without delay. ^102^

Some preexisting liver diseases are risk factors for poorer prognosis in COVID-19. Preexisting liver disease increases ACE2 expression on hepatocytes (observed in murine and human studies) and may thus increase the hepatic tropism of SARS-CoV-2. ^24^ Grasselli et al. found 3% of 1,591 COVID-19 patients admitted to ICUs had a history of chronic liver disease. ^105^

### Nonalcoholic fatty liver disease

Patients with NAFLD have a higher risk of COVID-19 progression (6.6% versus 44.7%) and a higher likelihood of abnormal LFTs (70% versus 11.1%). ^68^ It is a risk factor for hospitalization in COVID-19, and was suggested as a more significant factor than age, gender, obesity, or other comorbidities. ^112^ Although the underlying mechanism is yet to be clarified, a possible reason could be impaired innate immune responses to the virus. Hepatic macrophages/Kupffer cells may be skewed from an inflammatory-promoting to inflammatory-suppressing type. ^68^ Nonalcoholic fatty liver disease is associated with an increased risk of severe COVID-19, even after adjusting for obesity. ^113^ Zheng et al. found a 6-fold higher risk of severe COVID-19 in patients with NAFLD. The severity of COVID-19 was much higher in obese than nonobese NAFLD patients. ^114^ According to Prins et al., the liver contains the highest number of macrophages, and NAFLD patients often present with elevated cytokine levels. Nonalcoholic fatty liver disease progression could also be hastened by COVID‐19. ^115^

### Cirrhosis.

These patients are more susceptible to SARS-CoV-2 infections, owing to their immunocompromised status. Increased disease severity and complications lead to higher mortality. A multicenter study of 50 cirrhosis patients with COVID-19 found a 30-day mortality rate of 34%. ^116^ Higher severity of the underlying liver disease was associated with an increased risk of mortality in COVID-19. In a study of 152 COVID-19 patients with chronic liver disease (including 103 with cirrhosis), the mortality rate was 40%. The mortality rates in patients with Child-Pugh (CP) class A cirrhosis, CP class B cirrhosis, and CP class C cirrhosis were 24%, 43%, and 63% respectively. CP class B or C cirrhosis were independent predictors of mortality. ^117^

### Hepatocellular carcinoma.

Patients with hepatocellular carcinoma (HCC) often need to visit a hospital for their treatment (chemotherapy and or immunotherapy) and thus need to be managed and monitored carefully. They have a higher risk of getting hospital-associated COVID-19, especially those who underwent surgery or received systemic treatment in the prior month. Post-epatectomy liver failure (PHLF) is a life-threatening situation following hepatectomy. Increased inflammation during COVID-19 may predispose the patient to PHLF. Secretion of IL-6 after hepatectomy and during PHLF, may further increase inflammation during COVID-19. ^118^ In patients with HCC, COVID-19 may exacerbate existing chronic liver disease and complicate cancer management. Cancer patients have a higher risk of infection and worse outcomes, especially those who have recently undergone cancer treatment. Hepatocellular carcinoma was underrepresented in COVID-19 series. Mitigation measures should be implemented to minimize the exposure of such patients to the virus. A decision on the treatment of HCC should be balanced with the availability of medical resources and the level of risk of getting COVID-19. ^119^

### Hepatitis B infections.

Globally, there are over 250 million people living with HBV infection. ^120^

Thus, it is important to study the clinical characteristics of COVID-19 patients with preexisting HBV infection. These patients tend to have a more severe form of COVID-19. ^69^^,^ ^121^ However, according to the COVID-HBV-Chinese Portal Hypertension Diagnosis and Monitoring Study Group study, patients with preexisting HBV infections had a lower incidence of ICU admission or death. ^8^ Similar findings were noted with SARS-CoV-1 and HBV coinfection. ^122^ In a systematic review done by Hossein Mirzaie et al., the mortality was 6% in COVID-19-HBV, coinfected persons. The low ICU admission and death rates in preexisting HBV patients maybe because of host immune responses that result from indirect interactions between HBV and SARS-CoV-2 virus. ^8^ Furthermore, early COVID-19 vaccination in HBV-infected populations, additional precautionary measures, and early identification and treatment of COVID-19 infection may have contributed to better outcomes. During treatment, discontinuation of antiviral treatment of hepatitis B is discouraged, so as to prevent its reactivation. Anti-HBV drugs may be considered when patients are on immunosuppressive treatment and the patients should be monitored carefully. ^123^

### Alcoholic liver disease.

Both alcohol-associated liver disease (ALD) and alcohol use disorders (AUD) have been affected during the COVID-19 pandemic. Economic and social stresses resulting from the pandemic increased alcohol consumption in some individuals and delays in care have led to increased mortality from alcohol-associated hepatitis. ^117^

### Liver transplant recipients.

A meta-analysis comparing 1522 COVID-19-infected liver transplant (LT) patients and around 240,000 COVID-19-infected non-LT patients showed similar mortality rates. ^106^ The LT patients had a cumulative mortality rate of 17.4%. ^106^ The graft dysfunction rate was 2.3% (1.3–4.1%). However, 23% developed severe infections. ^106^ A review by Kullar et al. showed that 80% of LT patients with COVID-19 required hospital admission and 17% required intensive care. ^124^ Around 21% required mechanical ventilation and the overall mortality was 17%. ^124^ Therefore, these patients would require close monitoring during the active stage of infection with observation for graft rejection. Vaccination as a preventive strategy is recommended. Postexposure prophylaxis should be considered in selected high-risk individuals. Due to lack of consensus, management strategies varied widely, including variations in immunosuppressive therapy and different investigational therapies to manage COVID-19 in transplant patients. ^125^

### Surgical aspects.

Surgical services, especially routine surgeries for both benign and malignant conditions, have been affected worldwide during the COVID-19 pandemic. ^126^^–^ ^128^ The effect of surgery and anesthesia has negative implications on COVID-19 patients. Furthermore, healthcare workers were also affected because of increased high-risk exposures and the lack of clinical exposure/training due to the postponement of routine surgeries. ^129^^–^ ^131^ Surgery should not be delayed for HCC patients. However, adequate precautions should be taken to minimize complications, which include prior vaccination and proper timing if previously infected by SARS-CoV-2 and thromboprophylaxis. ^126^^,^ ^130^^,^ ^132^ Patients are especially vulnerable to pulmonary complications and venous thromboembolism and these should be prevented. ^133^^,^ ^134^ Routine preoperative screening for SARS-CoV-2 is mandatory and COVID-19-free pathways have been shown to be beneficial. ^135^^,^ ^136^ However, preoperative isolation is controversial. ^137^ During periods of societal restriction, the resilience of elective surgery systems requires strengthening to prevent postponement of cancer surgeries. ^138^^,^ ^139^

Liver transplantation has been affected due to COVID-19 infection worldwide. ^140^ Individuals with a liver transplant need preoperative, surgical intervention, and postoperative care, which is challenging during this pandemic. The healthcare facilities are overwhelmed with the management of COVID-19 and the need for resources such as ICU beds and ventilators. ^136^ In addition, to limited facilities, the exclusion of donors with COVID-19 is a major problem encountered in the transplantation programs. Furthermore, immunosuppressive therapy in transplanted individuals and drug interactions may make them more vulnerable for COVID-19 infection and hence optimal protective measures should be maintained. The European Association for the Study of the Liver (EASL) and AASLD have suggested that LT should not be postponed during the pandemic. Preliminary data has shown that despite immunosuppression in LT patients, no increased risk was found with post-LT patients. ^107^ This may suggest that immunosuppression in LT patients was not associated with an increased risk of COVID-19 infection. However, further studies are needed prior to this becoming routine clinical practice.

In Italy, though liver transplantation was carried out during COVID-19, a 25% reduction in procured organs was observed during the first 4 weeks of the outbreak. ^107^ Both living and deceased donor LTs were performed and increased mortality was seen in patients who needed to remain on the waiting list. ^141^ Saracco et al. found no significant difference in numbers of patients undergoing LT from deceased donors in 2020 compared with 2019. The rate of early graft dysfunction was 24% and 33% in 2020 and 2019. In 2020, the Median Model for End-stage Liver Disease (MELD) score was higher (17 versus 13) and there were no deaths in those on the waiting list. ^107^ Thus careful testing of symptomatic patients and careful testing all transplant donors for SARS-CoV-2 RNA and team-work helps overcome the constraints in LT during the COVID-19 pandemic. Furthermore, a patient’s liver transplantation candidacy should not be affected by PCR test results alone, as the COVID-19 PCR may remain positive in absence of active COVID-19. ^108^

### Drug interactions.

Drug–drug interaction is a problem associated with LT during the COVID-19 era. Patients who underwent LT are usually poly-medicated mainly with immunosuppressive drugs. Since a number of drugs used in COVID-19 have only been recently authorized, monitoring for potential drug interactions is important. ^141^ Remdesivir has been approved by US Food and Drug Administration (US FDA) for the treatment of hospitalized patients with COVID-19. It is potentially hepatotoxic and should be used with caution. Increased liver transaminase levels are a common adverse effect, and discontinuation of remdesivir infusions should be considered if elevations in ALT or AST above 10 times the upper limit of normal are noticed. Baseline LFTs should be done before initiation of therapy and these should be monitored closely during therapy. ^109^^,^ ^142^ The concentration of remdesivir may be affected by enzyme inducers such as clarithromycin, rifampin, phenytoin, and phenobarbital. ^143^ Favipiravir increases the concentration of pioglitazone, rosiglitazone, paracetamol, oseltamivir, and hormonal replacement therapy, but does not have significant interactions with immunosuppressive medications or steroids. ^143^^,^ ^144^ Paxlovid, an oral antiviral agent contains the protease inhibitor nirmatrelvir and a low dose of ritonavir. Ritonavir may cause liver injury and thus caution needs to be exercised when Paxlovid is considered for patients with liver enzyme abnormalities, hepatic inflammation, or preexisting liver diseases. ^145^ Tociluzumab has minor interactions with ciclosporin, tacrolimus, and sirolimus. It may also reduce the concentrations of calcineurin inhibitors and drug level monitoring should be performed. Its use with chloroquine and hydroxychloroquine may produce additive toxicity. Tocilizumab has a myelosuppressive effect and it may thus potentiate hematological toxicity of ribavirin and interferon-beta if used together. In the setting of LT, interferon-beta has no interactions with immunosuppressive drugs or steroids. However, as it induces myelosuppression, it should not be combined with tocilizumab. Also, potential interaction with chloroquine and hydroxychloroquine may increase its toxicity. ^141^

## COVID-19 VACCINES IN LIVER DISORDERS

Patients with CLD (predominantly cirrhosis), hepatobiliary malignancies, candidates for liver transplantation, and immunosuppressed individuals ^110^ after liver transplantation appear to be at increased risk of COVID-19 infection and increased mortality. This risk might occur through cirrhosis-associated immune dysfunction, acute hepatic decompensation, and a systemic inflammatory response. ^146^ Therefore, COVID-19 vaccines should be administered as early as possible to patients with CLD. In general, vaccines are less effective in CLD and post-LT patients. The impaired immune response in such patients may result in an incomplete immediate and long-term immune protection following vaccination. ^147^ The original vaccine trials included only small numbers of patients with mild to moderate liver disease and excluded those on immunosuppressive medications. ^148^ Chronic liver disease patients represented less than 0.5% of those enrolled in phase II clinical trials. From these clinical trial results, it was difficult to speculate which COVID-19 vaccine type would be most effective in those with CLD. Liver transplant individuals usually have reduced rates of seroconversion and lower antibody titers in response to vaccination and this may be similar with the COVID-19 vaccines. ^146^ In a recent US study, 63% of post-LT patients seroconverted after the second dose, whereas 100% of cirrhotic patients did so. Furthermore, 28% of LT patients did not develop humoral or T cell responses, pointing to the need for routine serological testing with third vaccine dose administration in such patients. ^149^ Strauss et al. found that LT patients who received two doses of an mRNA vaccine to have a greater antibody response than other solid organ transplants (SOT). This may be due to milder induction immunosuppression given to LT patients when compared with other SOT patients such as heart and lung transplant patients. ^150^ Early vaccination and avoiding the use of antimetabolite medications (if this were at all possible) should be considered for obtaining better postvaccine immune outcomes in these patients. A third dose of the COVID-19 vaccine needs to be considered for LT recipients, at around 1 or 2 months after their second dose. In patients with CLD, vaccination may not result in a robust immune response due to immunosuppression. ^151^ Hence, monoclonal antibody therapy may be beneficial in these patients. Vaccine-related adverse effects in LT recipients are similar to other individuals. ^152^

## LIMITATIONS

A limitation of this review is that the majority of studies are observational and have small numbers of subjects making it difficult to provide more definitive conclusions. It is possible that subtle liver findings were not documented (and thus underestimated) during the early part of the pandemic. Well-conducted studies from different regions of the world would help expand the evidence base and provide better answers to the many questions at hand. Ours is a broad overview of the main reported hepatic manifestations in COVID-19 and their management. A more comprehensive and detailed profile of specific aspects should emerge as more data are published from different countries.

## CONCLUSION

In conclusion, liver involvement is observed in COVID-19 patients and may influence disease prognosis and outcomes. The factors that may contribute to liver involvement in COVID-19 include direct viral cytopathic effects, exaggerated immune responses, hypoxia-induced changes, vascular changes due to coagulopathy, endothelitis, cardiac congestion from right heart failure, and drug-induced liver injury. Further clinical and laboratory studies should help ascertain more details on the potential mechanisms of SARS-CoV-2 infections and the liver. The COVID-19 vaccines should be administered as early as possible to patients with CLD and a third dose of the vaccine needs to be considered for LT recipients.
